# Species Richness-Environment Relationships of European Arthropods at Two Spatial Grains: Habitats and Countries

**DOI:** 10.1371/journal.pone.0045875

**Published:** 2012-09-24

**Authors:** Martin H. Entling, Oliver Schweiger, Sven Bacher, Xavier Espadaler, Thomas Hickler, Sabrina Kumschick, Ben A. Woodcock, Wolfgang Nentwig

**Affiliations:** 1 Institute for Environmental Sciences, University of Koblenz-Landau, Landau/Pfalz, Germany; 2 Community Ecology, University of Bern, Bern, Switzerland; 3 UFZ, Helmholtz Centre for Environmental Research, Department of Community Ecology, Halle, Germany; 4 Ecology and Evolution Unit, University of Fribourg, Fribourg, Switzerland; 5 Animal Biodiversity Group, Ecology Unit and CREAF, Autonomous University of Barcelona, Bellaterra, Spain; 6 Biodiversity and Climate Research Centre (BiK-F) & Senckenberg Gesellschaft für Naturforschung & Department of Physical Geography at Goethe University, Frankfurt/Main, Germany; 7 Centre of Excellence for Invasion Biology, Department of Botany and Zoology, Stellenbosch University, Matieland, South Africa; 8 NERC Centre for Ecology & Hydrology, Wallingford, Oxfordshire, United Kingdom; University Copenhagen, Denmark

## Abstract

We study how species richness of arthropods relates to theories concerning net primary productivity, ambient energy, water-energy dynamics and spatial environmental heterogeneity. We use two datasets of arthropod richness with similar spatial extents (Scandinavia to Mediterranean), but contrasting spatial grain (local habitat and country). Samples of ground-dwelling spiders, beetles, bugs and ants were collected from 32 paired habitats at 16 locations across Europe. Species richness of these taxonomic groups was also determined for 25 European countries based on the Fauna Europaea database. We tested effects of net primary productivity (*NPP*), annual mean temperature (*T*), annual rainfall (*R*) and potential evapotranspiration of the coldest month (*PET_min_*) on species richness and turnover. Spatial environmental heterogeneity within countries was considered by including the ranges of *NPP*, *T*, *R* and *PET_min_*. At the local habitat grain, relationships between species richness and environmental variables differed strongly between taxa and trophic groups. However, species turnover across locations was strongly correlated with differences in *T*. At the country grain, species richness was significantly correlated with environmental variables from all four theories. In particular, species richness within countries increased strongly with spatial heterogeneity in *T*. The importance of spatial heterogeneity in *T* for both species turnover across locations and for species richness within countries suggests that the temperature niche is an important determinant of arthropod diversity. We suggest that, unless climatic heterogeneity is constant across sampling units, coarse-grained studies should always account for environmental heterogeneity as a predictor of arthropod species richness, just as studies with variable area of sampling units routinely consider area.

## Introduction

Since the early observations of Darwin and von Humboldt, ecologists have attempted to explain why species diversity increases towards the Equator. Relationships of biodiversity with net primary productivity (*NPP*) are frequently suggested as potential explanations [1]–[4], and *NPP* is usually the best correlate of biodiversity [5]. Productivity-diversity relationships are assumed to be driven by the *NPP* of an ecosystem as a result of increased provision of vital resources [1]. For example, climates which are highly deficient in water (such as desert) or energy (such as arctic) have both low *NPP* and low species richness. There is, however, little consensus on the mechanisms underpinning increases in diversity from intermediate to high levels of *NPP* [2], [6]. Furthermore, the shape of productivity-diversity relationships is typically dependent on the spatial grain of the analysis. For example, linear increases in species richness in large sampling units (such as degree grids) contrast with the multiple ways in which species richness within local habitats responds to *NPP* [7]–[9]. Thus, although productivity-diversity relationships are widespread [5], the underlying mechanisms still need to be resolved [10].

In other cases however, biodiversity is more strongly correlated to ambient energy than to *NPP* [11]–[14]. In contrast to *NPP*, ambient energy does not include water availability and can be expressed as annual mean temperature (*T*). Possible mechanisms for increasing species richness with ambient energy include tropical niche conservatism [13], dispersal limitation after glaciation [14] and metabolic theory [11], [15]. Based on the evolutionary origin of many taxa in tropical climates, their occurrence in cooler climates depends on the evolution of cold-tolerance. Within larger taxonomic groups, communities in warm climates often include many basal taxa, while communities in temperate to cold climates are increasingly restricted to few derived taxa. This results in a positive relationship of species richness and *T*. Analyses of the phylogenetic structure of communities provide evidence for this mechanism, notably in butterflies [13]. Another effect of historical climate on contemporary richness patterns has been described for European dung beetles [14]. Their limit of thermal tolerance during the last glacial maximum marks a strong change in current richness with low species numbers north of this limit. Thus, limited colonization of areas with historically unsuitable climate can contribute to current correlations between richness and ambient energy. Metabolic theory provides an additional mechanism for higher species richness in warmer climates based on the influence of temperature on metabolic rates and on rates of speciation [11]. Metabolic theory predicts a direct, monotonic relationship of species richness with *T*, whereby the slope of log_e_ –transformed species richness with inverse *T* is predicted to be −0.65 [15].

A third framework for broad-scale patterns of species richness is biological relativity to water-energy dynamics. It is based on the dependence of all life on the availability of water in a liquid form [16]–[17]. This framework suggests that species richness is proportional to (a) the availability of liquid water (annual rainfall, *R*), and (b) the lowest monthly value of potential evapotranspiration (*PET_min_*). This “interim general model” explains almost 80% of the variation in species richness of trees and shrubs in eastern and southern Africa [16]. At higher latitudes (Europe and North America) the effect of rainfall on species richness of trees dominates (*r* = 0.64), and effects of *PET_min_* or other energy variables are non-significant [18].

The final determinant of species richness patterns considered here is spatial environmental heterogeneity. Its importance can be expected to increase with increasing size of sampling units (spatial grain [2], [19]). For example, only limited spatial climatic heterogeneity can be expected along a 21 m transect within one habitat type (the small sampling grain in our study, see Methods). In contrast, large differences in internal spatial climatic heterogeneity exist among countries (the large sampling grain in our study). For example, *T* differs by 13.3°C between the warmest and the coldest 10×10 arc minute square within Switzerland – more than ten times the difference within Denmark, which has a similar surface area (Table 1). Effects of spatial environmental heterogeneity on species richness can be explained with niche theory, which assumes different environmental preferences and tolerances among species [20], [21]. Among environmental variables, climate is central to the distribution and persistence of species worldwide [22]. In particular, the distribution of numerous taxa is influenced by *T*, for example plants [23], beetles [14], [24], spiders [25] and birds [26]. Thus, heterogeneity of environmental conditions (such as *T*) within large sampling units can enhance overall species richness by providing suitable conditions for larger numbers of species with different ecological niches. Increased species richness due to an increased importance of spatial environmental heterogeneity will consequently shift the focus from alpha diversity (local species richness) to beta diversity (turnover of species in space [27]).

**Table 1:** The *N* = 25 countries included in the analyses with environmental variables and species richness.

Country	Area	*NPP*	*NPP_range_*	*T*	*T_range_*	*R*	*R_range_*	*PET_min_*	*PET_minrange_*	*S_a_*	*S_c_*	*S_h_*	*S_f_*	*S_her_*	*S_car_*
Austria	83.9	0.61	0.58	6.5	12.6	870	962	0.0	2.2	972	2980	1470	122	3388	2156
Belgium	30.5	0.59	0.10	9.8	2.7	886	529	7.8	11.8	690	1570	946	70	2168	1108
Bulgaria	110.9	0.56	0.22	10.4	10.6	580	209	0.6	6.3	947	2430	1501	154	2993	2039
Czech Republic	78.9	0.61	0.16	7.8	5.3	550	470	0.0	0.0	842	2356	1390	117	2802	1903
Denmark	43.1	0.55	0.06	8.2	1.2	693	352	3.1	5.8	505	1519	814	58	1791	1105
Estonia	45.1	0.52	0.07	5.4	1.8	486	136	0.0	0.0	503	1157	669	47	1464	912
Finland	338.2	0.46	0.32	1.3	9.9	353	261	0.0	0.0	623	1345	810	58	1865	971
France	542.8	0.65	0.35	10.8	16.7	837	817	10.1	22.2	1415	4226	2050	218	4967	2942
Germany	357.0	0.59	0.13	8.8	6.8	690	625	1.9	9.2	1032	2628	1500	129	3219	2070
Great Britain	242.9	0.48	0.33	8.6	6.8	1164	2932	13.6	22.1	652	1687	936	66	2117	1224
Greece	115.4	0.49	0.37	13.5	11.2	665	873	8.4	20.6	619	2463	1552	209	2890	1953
Hungary	93.0	0.59	0.17	10.7	3.4	543	299	0.0	0.4	740	2479	1257	89	2660	1905
Italy	251.5	0.60	0.71	11.7	19.8	844	1316	7.8	21.7	1374	4742	1929	213	5180	3078
Latvia	64.6	0.55	0.07	6.0	2.4	509	230	0.0	0.0	399	1175	699	44	1409	908
Lithuania	65.2	0.58	0.10	6.5	1.6	523	197	0.0	0.0	382	907	540	50	1187	692
The Netherlands	41.5	0.55	0.07	9.7	1.4	772	124	9.3	5.0	611	1562	967	67	1980	1227
Norway	323.9	0.41	0.56	1.3	12.3	613	3173	0.2	10.5	567	1231	706	47	1671	880
Poland	323.3	0.61	0.14	8.1	6.4	504	509	0.0	0.0	793	2370	1213	94	2629	1841
Portugal	92.0	0.53	0.41	15.2	6.9	873	1235	19.1	19.1	651	1417	930	125	2100	1023
Romania	238.4	0.56	0.33	9.2	9.9	553	489	0.0	3.5	965	2231	1290	118	2944	1660
former Serbia and Montenegro	102.2	0.63	0.18	10.2	12.0	754	1109	0.7	9.9	685	1709	1217	213	2367	1457
Slovakia	49.0	0.64	0.12	8.1	7.2	624	427	0.0	0.0	898	2501	1194	85	2845	1833
Spain	499.8	0.53	0.46	13.2	15.8	636	1628	12.2	26.7	1194	3623	1809	285	4334	2577
Sweden	450.0	0.47	0.56	2.0	13.9	407	642	0.0	1.5	725	1705	972	71	2211	1262
Switzerland	41.3	0.59	0.66	5.4	13.3	968	776	0.3	4.5	941	2308	1225	137	2959	1652

Environmental variables: area (in 10^3^ km^2^, islands excluded), averages of net primary productivity (*NPP*; in kg C per m^2^ per year) and annual mean temperature (*T*; in °C), annual rainfall (*R*; in mm) and minimal potential evapotranspiration (*PET_min_*; in mm), and the spatial heterogeneity in primary productivity (*NPP_range_*), annual mean temperature (*T_range_*), annual rainfall (*R_range_*), minimal potential evapotranspiration (*PET_minrange_*). Species richness for spiders (*S_a_*), beetles (*S_c_*), bugs (*S_h_*), ants (*S_f_*), herbivores (*S_her_*) and carnivores (*S_car_*).

Here, we explore species richness-environment relationships of European arthropods. We combine the results of a continent-wide standardised sampling programme of local ground-dwelling arthropod communities (local grain) with existing coarse-grained country inventories (country grain) of comparable spatial extent. At the local grain ground-dwelling ants (Formicidae), beetles (Coleoptera), bugs (Hemiptera) and spiders (Araneae) were sampled in 32 habitats at 16 locations across Europe, ranging from boreal to Mediterranean in climate. At the country grain, inventories of 25 European countries were obtained for the same groups [28]. We used these data to test each of the above hypotheses by first comparing the explanatory power of productivity, ambient energy, the interim general model, and the best possible statistical model (drawn from all variables) for biodiversity within local habitats (alpha diversity). Secondly, we compared the potential for environmental heterogeneity to explain species turnover across locations (beta diversity). Thirdly, we compared the explanatory power of productivity, ambient energy, the interim general model, spatial environmental heterogeneity and the best possible statistical model (drawn from all variables) for biodiversity within countries (gamma diversity).

## Methods

### Ethics

Field sites were selected and established within the EU FP6 ALARM project to form a long-lasting research network
Each field site had a site manager, responsible for contacts to local authorities and/or land owners
In most cases the land belonged to regional research stations
More detailed descriptions can be found in reference [29]
Protected areas or rare habitats were not included into this field site network
We did not include protected species.

The employed pitfall traps capture invertebrates, with no protected species affected in the habitats we sampled. No permissions are needed to use pitfall traps outside of protected areas.

### Data

**Figure 1:**
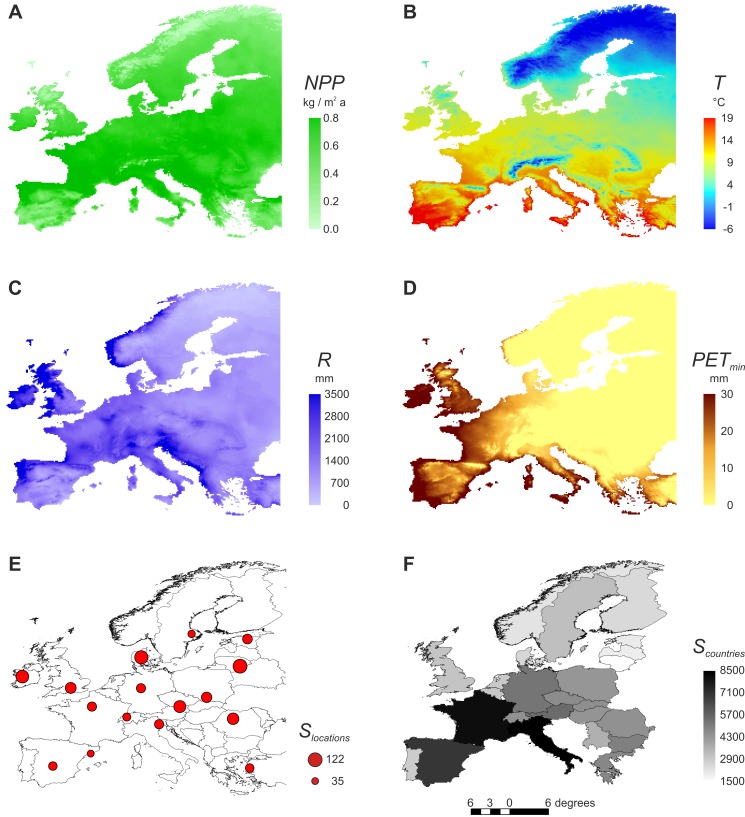
Map of Europe showing the studied environmental variables. (A) *NPP* = net primary productivity, (B) *T* = annual mean temperature, (C) *R* = annual rainfall, and (D) *PET_min_* = potential evapotranspiration of the coldest month, plus (E) the *N* = 16 study locations with their species richness *S_locations_* (all groups combined; average between natural and disturbed habitat) and (F) the *N* = 25 analysed countries with their species richness *S_countries_* (all groups combined).

Ground-dwelling arthropods were sampled in 32 habitats at 16 locations across Europe (Table 2, Fig. 1E). Thirteen locations were part of the ALARM field site network [30], and three sites (Bern, Silkeborg, Wien) were added to fill geographic gaps. As far as possible, one forest as an example of a near-natural habitat and one cereal field as an example of an intensive agricultural habitat were sampled in each location. When unavailable, other near-natural habitats (scrubland or extensive grassland) and other intensive agricultural habitats (intensive grassland or olive grove) were sampled instead (Table 2). Trapping took place in 2006 and started five days after the beginning of the vegetation period (the onset of growth in the majority of plant species) in each location [31]. In each habitat, eight pitfall traps of 7 cm diameter were placed along a transect and separated by 3 m from each other. The traps were filled with 0.1 L of a 4% formaldehyde solution, to which sodium dodecyl sulphate was added as detergent. Three sampling periods of two weeks were separated by pauses of two weeks. Adult arthropods were identified to species level by specialists. The following groups were considered: ants (Hymenoptera: Formicidae), beetles (Coleoptera: Carabidae, Curculionoidea, and Staphylinidae), bugs (Hemiptera: Auchenorrhyncha and Heteroptera), and spiders (Araneae). In addition to the analysis of taxonomic groups, we divided the studied arthropods into trophic groups according to the dominant feeding type in the respective family. Herbivore families were all Curculionoidea, all Auchenorrhyncha and the heteropteran families Berytidae, Cydnidae, Lygaeidae, Miridae, Pentatomidae, Piesmatidae, Plataspidae, Pyrrhocoridae, Rhopalidae, Scutelleridae and Tingidae. Spiders, ants and the remaining beetle and bug families were carnivores. Detritivores could not be analysed because this feeding type did not dominate in any of the sampled families. Species numbers within 25 European countries were taken from the Fauna Europaea database [28] (Table 1, Fig. 1F). Countries smaller than 30,000 km^2^ were excluded, as were all countries for which the known number of arthropod species lay below the 95% confidence interval of the species - log area relationship, indicating effects of insularity (Republic of Ireland) or incomplete knowledge of the arthropod fauna (Ukraine, Belarus). We further excluded islands such as the Balearic Islands, Corsica, Greek Islands, Sardinia and Sicily from the respective mainland areas.

**Table 2:** The *N* = 16 sampling locations with geographic coordinates, environmental variables, species richness and individual numbers.

Location	°N	°E	*NPP*	*T*	*R*	*PET_min_*	Natural habitat													Disturbed habitat												
							type	*S_a_*	*S_c_*	*S_h_*	*S_f_*	*S_her_*	*S_car_*	*N_a_*	*N_c_*	*N_h_*	*N_f_*	*N_her_*	*N_car_*	type	*S_a_*	*S_c_*	*S_h_*	*S_f_*	*S_her_*	*S_car_*	*N_a_*	*N_c_*	*N_h_*	*N_f_*	*N_her_*	*N_car_*
Berkshire (United Kingdom)	51.5	−1.3	0.53	9.6	726	13.7	scrub	63	47	5	9	19	105	896	201	17	842	88	1868	cereal	28	35	5	1	5	64	298	588	13	3	11	891
Bern (Switzerland)	47.0	7.4	0.63	8.4	888	0.0	forest	30	30	1	2	5	58	199	314	2	19	12	522	cereal	23	41	3	1	8	60	763	1219	6	19	30	1977
Cluj (Romania)	46.7	23.9	0.56	9.1	517	0.0	scrub	48	41	20	14	32	91	255	113	37	694	59	1040	cereal	31	13	15	12	19	52	96	29	20	142	26	261
Galway (Republic of Ireland)	53.2	−8.8	0.55	9.8	1066	18.9	scrub	41	67	16	4	29	98	291	556	46	52	259	686	grass	31	61	5	1	16	82	201	542	5	15	129	634
Garraf (Spain)	41.3	1.8	0.60	15.1	611	18.6	forest	14	3	2	7	2	24	67	5	2	58	4	128	cereal	27	4	8	4	6	37	114	5	25	295	15	424
Göttingen (Germany)	51.4	9.6	0.57	8.7	749	1.1	forest	30	38	0	0	11	57	176	511	0	0	44	643	cereal	27	40	3	0	6	64	377	317	12	0	19	687
Île-de-France (France)	48.6	2.0	0.61	11.0	625	11.4	forest	22	30	6	6	9	55	180	178	12	110	23	457	cereal	26	45	3	0	6	68	278	1407	4	0	51	1638
Kraków (Poland)	50.0	19.8	0.66	8.3	578	0.0	scrub	43	26	9	7	17	68	237	392	13	781	350	1073	grass	41	44	13	3	26	75	467	185	105	144	133	768
Lesvos (Greece)	39.2	26.5	0.39	16.2	633	13.8	grass	27	5	8	17	10	47	145	7	29	820	31	970	olive	33	21	14	11	19	59	143	92	44	1199	45	1433
Meolo (Italy)	45.9	12.4	0.64	13.1	1107	5.5	grass	20	23	5	10	5	53	52	87	6	50	6	189	grass	28	28	16	8	20	60	131	117	62	943	68	1185
Silkeborg (Denmark)	56.2	9.7	0.55	7.7	674	1.1	forest	54	42	10	4	16	93	612	324	17	67	166	854	cereal	40	69	8	3	14	106	460	974	36	16	66	1420
Tartu (Estonia)	59.1	26.2	0.51	5.0	485	0.0	forest	23	32	3	2	11	49	189	182	3	4	38	340	cereal	24	44	11	0	17	62	378	1096	26	0	39	1461
Toledo (Spain)	39.4	−4.0	0.48	13.4	527	9.2	scrub	25	14	16	24	18	61	128	50	141	1799	143	1975	cereal	24	16	8	9	10	47	133	69	16	358	20	556
Uppsala (Sweden)	59.8	17.5	0.53	5.8	403	0.0	scrub	25	26	19	9	21	58	141	124	84	266	86	529	cereal	16	20	5	1	7	35	327	220	9	1	9	548
Vilnius (Lithuania)	54.8	25.0	0.55	6.5	519	0.0	forest	46	41	12	4	12	91	552	246	29	497	29	1295	cereal	29	89	18	4	34	106	806	2401	140	46	135	3258
Wien (Austria)	48.6	15.7	0.64	8.7	543	0.0	forest	30	46	3	9	9	79	193	386	5	247	19	812	cereal	30	58	16	6	15	95	887	837	37	16	36	1741

Environmental variables: primary productivity (*NPP*; in kg C per m^2^ per year), annual mean temperature (*T*; in °C), annual rainfall (*R*; in mm), minimal potential evapotranspiration (*PET_min_*; in mm). Species richness (*S*) and individual numbers (*N*) per habitat with subscripts as in Table 1.

Environmental variables were extracted from a European gridded data set with a monthly time step and a spatial resolution of 10×10 arc minutes, which corresponds approximately to 16 km [32]. The primary climatic variables temperature (*T*, Fig. 1B) and precipitation were constructed through interpolation from station observations [33]. Annual rainfall (*R*; Fig. 1C) was calculated as the sum of precipitation in all months with an average temperature >0°C [18]. *NPP* (Fig. 1A) was estimated by running the LPJ-GUESS ecosystem model [34], [35] with the same climate data, and parameterized for the potential natural vegetation of Europe [36], [37]. LPJ-GUESS and the closely related LPJ-DGVM [34] have formerly been shown to reproduce observed variations in *NPP* across various types of vegetation and climates [38]–[40]. *PET_min_* (Fig. 1D) represents potential evapotranspiration of the coldest month of each year, and was calculated using the Thornthwaite equation, which only requires knowledge of air temperature [41]. We used long-term annual means of *NPP*, *T*, *R* and *PET_min_* from 1971 to 2000. For analysing species richness within habitats and species turnover across habitats, we used *NPP*, *T*, *R* and *PET_min_* values of the grid in which the habitats were located. For analysing species richness within countries, explanatory variables were averaged across all grids of the country. In addition, spatial heterogeneity in *NPP*, *T*, *R* and *PET_min_* were calculated as ranges for each country by subtracting the minimal from the maximal value, respectively (i.e. difference between the grid cells with the highest and lowest value).

### Analysis

Local species richness and species richness in European countries was analysed using linear models with standardised explanatory terms (mean = zero, standard deviation = 1) in the statistical environment R version 2.12.0 [42]. To account for possible differences in sampling efficiency between locations, we used the number of captured individuals *N* as a covariate in the analyses of local species richness. We accounted for possible effects of spatial autocorrelation of the habitats within the locations and among the locations and countries with generalised least squares [43] with spatial simultaneous autoregressive error models [44]. Models were based on neighbourhood matrices that linked the two habitats within a location and each location with at least one other location for the local grain analyses and allowed each country to be in the neighbourhood of at least one other country, i.e. at a maximum distance of about 850 km from centre to centre. For this we used the package *spdep* [45]. In addition, we calculated Moran’s *I* correlograms for the residuals of models with and without correction for spatial autocorrelation to assess if the tested theories miss important spatially structured environmental variables. Missing crucial spatially structured environmental variables will lead to significant residual spatial autocorrelation of the uncorrected models.

Separate models of local species richness were calculated according to productivity-diversity relationships, ambient energy and the interim general model, plus one “Best” model in which all explanatory variables relevant for the different theories (*NPP*, *NPP* ^2^, *T*, *R*, *PET_min_* and *PET_min_* ^2^) entered the initial models. We included interactions of all linear terms with habitat to test if there are different responses in the different habitat types. We identified the minimal adequate models by a backwards variable selection procedure according to the second order Akaike information criterion (*AICc*). Linear terms were always kept in the model when the respective quadratic term increased the model fit. In cases of high collinearity (Pearsson *r* >0.5) of linear terms (see Tables 3, 4) we calculated separate models always containing only one of these terms, and the best model was chosen based on the *AICc* model selection criteria. This restricted the models to only one energy variable (either *T* or *PET_min_*). It also reduced the risk of overfitting, which is considerable given the low numbers of replicates (*N* = 16 locations and *N* = 25 countries).

**Table 3:** Pearson’s correlation coefficients between explanatory variables among the *N* = 16 locations.

	*NPP*	*T*	*R*
*T*	**−**0.23		
*R*	0.29	0.28	
*PET_min_*	**−**0.28	0.71	0.35

For abbreviations see Table 2.

**Table 4:** Pearson’s correlation coefficients between explanatory variables among the *N* = 25 countries.

	Area	*NPP*	*T*	*R*	*PET_min_*	*NPP_range_*	*T_range_*	*R_range_*
*NPP*	**−**0.27							
*T*	**−**0.07	0.40						
*R*	**−**0.17	0.21	0.41					
*PET_min_*	0.12	**−**0.13	0.67	0.61				
*NPP_range_*	0.44	**−**0.25	**−**0.08	0.31	0.19			
*T_range_*	0.64	0.00	0.07	0.18	0.14	0.84		
*R_range_*	0.43	**−**0.45	**−**0.02	0.49	0.40	0.54	0.42	
*PET_minrange_*	0.39	**−**0.12	0.61	0.59	0.83	0.42	0.51	0.60

For abbreviations see Table 1. Area was log_10_−transformed prior to the analysis.

As relationships of species richness with *NPP* can be either linear or hump-shaped [3], [7], [10], we allowed the quadratic term of *NPP* to remain in the productivity-diversity relationship models if that resulted in lower *AICc* values. Ambient energy models were calculated using untransformed species richness and *T*. To test predictions made by metabolic theory, we calculated the slope of log_e_(species richness) with 1/[0.0000862(273+*T*)] for comparison with the predicted slope of −0.65 [15], [46]. We used the first version of the interim general model (IGM1), where species richness is explained by a linear term of *R* plus a linear and quadratic term of *PET_min_* [16]. At the country grain, *R* and *PET_min_* had a Pearson correlation coefficient of 0.6. Nevertheless, we also tested the full model including both variables for means of completeness. We also performed an influence analysis using Cook’s distance. If data points had a Cook’s distance >0.5, indicating disproportional weight in the regression analysis, then the effect of excluding those data points from the model was examined.

Relationships of species turnover with environmental variables were analysed using Mantel tests [47]. We used presence-absence data of the trapped species per location (natural and disturbed habitat combined). Community dissimilarities were calculated as Morisita-Horn distances and related to Euclidean environmental distances between all possible pairs of sites. Each environmental variable was tested separately, and separate tests were calculated for spiders, beetles, bugs, ants, all groups combined, herbivores and carnivores using the function *mantel* in the package *vegan* (default settings [48]). The significance was based on Monte Carlo tests with 999 permutations.

Species richness of arthropods in European countries was analysed in a similar way as local species richness, with the following additions. We corrected for the area of the countries by including log_10_(area) as an additional explanatory variable in all models [49]. In addition to productivity-diversity relationships, ambient energy and interim general models, we calculated a model containing spatial environmental heterogeneity. Variables considered were the ranges of *NPP*, *T*, *R* and *PET_min_*. However, all these range variables were highly intercorrelated (Table 4) and thus it was not possible to include them simultaneously in one model. Therefore, we calculated separate models always containing one of these terms and selected the best model according to *AICc*. Again, we calculated “Best” models in which all explanatory variables relevant for the different theories entered the set of initial models containing only one of the range variables characterising spatial environmental heterogeneity.

## Results

### Species Richness in Local Habitats

**Figure 2:**
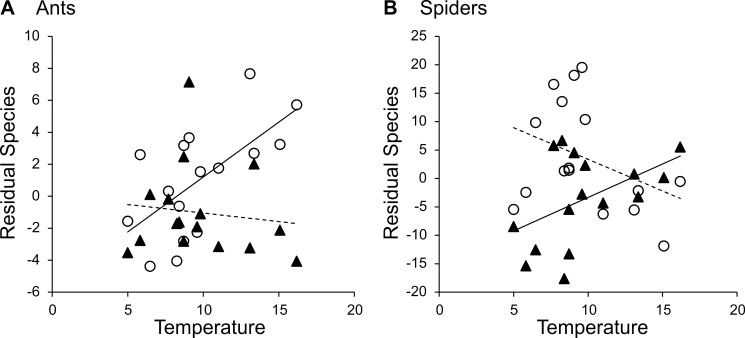
Interactive effects of *T* on species richness of (A) ants and (B) spiders in *N* = 16 near-natural (open circles) and *N* = 16 intensive agricultural (filled triangles) habitats. Residuals are from models of species richness corrected for the number of individuals captured in the respective habitat *N* and, in ants, for *R* (“Best” model in Table 5).

The samples contained 33223 individuals of our focal taxa that comprised 83 ant species (Formicidae), 444 beetle species (Coleoptera), 185 bug species (Hemiptera) and 354 spider species (Araneae). Relationships between species richness and environmental variables at the local grain were highly variable (Table 5). All arthropods combined and carnivores considered separately showed a hump-shaped relationship with *NPP*, while lacking a significant effect of ambient energy or variables from the interim general model. The ambient energy model was best for spiders, while beetles conformed most to the energy term of the interim general model. Bugs and herbivores were not significantly affected by any environmental variable. Ants showed a negative relationship to *R* plus an interactive effect of *T* and habitat type. Ant species richness increased with *T* in near-natural habitats, but did not change significantly with *T* in intensive agriculture (Fig. 2A). A more detailed examination of the response of ants to climate and habitat type has been given elsewhere [50]. Habitat type had a significant effect only on spiders, with higher species richness in near-natural than in intensive agricultural habitats (Table 5). In contrast to ants, there was a significant interactive effect of *T* and habitat type on spider richness, whereby species richness increased with *T* in agricultural habitats but did not significantly change with *T* in near-natural habitats (Fig. 2B). With respect to metabolic theory, only ants in near-natural habitats and spiders in intensive agricultural habitats had negative slopes of log_e_ (species richness) with *T* ^−1^ of −0.47±0.56 95% confidence interval (CI) and −0.43±0.27 95% CI, respectively. In all other cases, the slopes were positive and differed significantly from the predicted value of −0.65 (all arthropods: 0.20±0.18 95% CI; beetles: 0.12±0.30 95% CI; bugs: 0.05±0.21 95% CI; herbivores: 0.15±0.28 95% CI; carnivores: 0.21±0.17 95% CI; ants in intensive agricultural habitats: 0.22±0.43 95% CI; spiders in near-natural habitats: 0.12±0.55 95% CI).

**Table 5:** Models of species richness in *N* = 32 habitats at 16 locations across Europe.

	Group	*I*-lm	*I*-sar	*AICc*	*r* ^2^	*N*	*NPP*	*NPP* ^2^	*T*	*R*	*PET_min_*	*PET_min_* ^2^	Habitat	Habitat* *NPP*	Habitat* *T*	Habitat* *R*	Habitat* *PET_min_*
PDR	All	0.20**	0.02	288.85	0.58	18.48***	116.05**	−109.77**	NA	NA	NA	NA			NA	NA	NA
	Spiders	0.06	0.01	236.71	0.42	6.61***			NA	NA	NA	NA	7.95**		NA	NA	NA
	Beetles	−0.09	−0.01	251.23	0.7	16.72***			NA	NA	NA	NA			NA	NA	NA
	Bugs	−0.02	0.01	165.97	0.77	5.15***			NA	NA	NA	NA			NA	NA	NA
	Ants	0.01	−0.02	177.25	0.66	4.21***	−1.27*		NA	NA	NA	NA			NA	NA	NA
	Herbi	0.05	0.01	207.00	0.57	5.91***			NA	NA	NA	NA			NA	NA	NA
	Carni	0.19**	0.01	275.59	0.60	15.53***	83.30**	−77.92*	NA	NA	NA	NA	8.42		NA	NA	NA
AE	All	0.12*	0.01	290.65	0.47	17.43***	NA	NA		NA	NA	NA		NA		NA	NA
	Spiders	0.12*	0.02	233.82	0.57	8.21***	NA	NA	6.22**	NA	NA	NA	8.90***	NA	−6.95**	NA	NA
	Beetles	−0.09	−0.01	251.23	0.70	16.72***	NA	NA		NA	NA	NA		NA		NA	NA
	Bugs	−0.02	0.01	165.97	0.77	5.15***	NA	NA		NA	NA	NA		NA		NA	NA
	Ants	−0.02	0.01	176.05	0.73	3.99***	NA	NA	−0.55	NA	NA	NA	1.42	NA	3.21**	NA	NA
	Herbi	0.06	−0.01	206.97	0.57	5.90***	NA	NA		NA	NA	NA		NA		NA	NA
	Carni	0.18**	0.01	277.30	0.49	14.61	NA	NA		NA	NA	NA	8.90	NA		NA	NA
IGM	All	0.12*	0.01	290.65	0.47	17.43***	NA	NA	NA					NA	NA		
	Spiders	0.06	0.01	236.71	0.42	6.61***	NA	NA	NA				7.95**	NA	NA		
	Beetles	−0.14	0.01	247.34	0.78	17.23***	NA	NA	NA		−15.90**	18.86**		NA	NA		
	Bugs	−0.02	0.01	165.97	0.77	5.15***	NA	NA	NA					NA	NA		
	Ants	−0.03	0.01	179.48	0.60	4.59***	NA	NA	NA					NA	NA		
	Herbi	0.06	−0.01	206.97	0.57	5.90***	NA	NA	NA					NA	NA		
	Carni	0.18**	0.01	277.30	0.49	14.61	NA	NA	NA				8.90	NA	NA		
Best	All	0.20**	0.02	288.85	0.58	18.48***	116.05**	−109.77**									
	Spiders	0.12*	0.02	233.82	0.57	8.21***			6.22**				8.90***		−6.95**		
	Beetles	−0.14	0.01	247.34	0.78	17.23***					−15.90**	18.86**					
	Bugs	−0.02	0.01	165.97	0.77	5.15***											
	Ants	−0.07	−0.03	172.21	0.79	3.76***			0.07	−1.26**			1.54		3.04**		
	Herbi	0.06	−0.01	206.97	0.57	5.90***											
	Carni	0.19**	0.01	275.59	0.60	15.53***	83.30**	−77.92*					8.42				

Abbreviations: PDR = productivity-diversity relationships, AE = ambient energy, IGM = interim general model, Herbi = herbivores, Carni = carnivores. “NA” indicates cells that were excluded from the respective models. Empty cells were considered, but the respective explanatory variable did not remain in the model according to the second order Akaike Information Criterion (*AICc*). *I*-lm denotes Moran’s *I* correlation coefficient for the residuals of a linear regression model not corrected for spatial autocorrelation. *I*-sar denotes the respective Moran’s *I* for spatial autoregressive error models. *AICc* and *r* ^2^ values are from the spatial regression models, whereby *r* ^2^ is Nagelkerke’s pseudo *r* ^2^ based on maximum likelihood. *N* is the number of individuals captured at each location. Values in the columns of environmental variables are model coefficients, with significance levels denoted by asterisks:

* *p*<0.05, **p<0.01, ***p<0.001.

Interaction terms of Habitat*T indicate the difference in the slope for near-natural habitats compared to those for intensive agricultural habitats provided in *T*.

### Species Turnover

Species turnover across the 16 locations was most strongly correlated with differences in *T* (Table 6). Correlations were highest for spiders, carnivores, beetles and all groups combined, followed by ants and herbivores. Only bugs showed no significant relationship of species turnover with environmental variables. In the remaining groups, correlations of species turnover with differences in *T* were at least 49% stronger than with any other environmental variable. Correlations of species turnover with differences in *NPP* and/or *PET_min_* were significant, but substantially less strong than those with differences in *T*.

**Table 6:** Relationships of species turnover with differences in environmental variables across *N* = 16 locations (*N* = 15 locations for ants, because no ants were sampled in Göttingen).

Group	*NPP*	*T*	*R*	*PET_min_*
All Groups	0.40*	0.66***	−0.07	0.28*
Spiders	0.41*	0.69**	−0.05	0.29*
Beetles	0.40*	0.59***	−0.10	0.27(*)
Bugs	0.13	0.13	0.12	0.16(*)
Ants	0.28*	0.48**	−0.15	0.25*
Herbivores	0.11	0.33**	0.04	0.16(*)
Carnivores	0.41*	0.67***	−0.08	0.29*

Displayed are Mantel statistics (*r*) with significance levels based on 999 permutations and denoted by asterisks:

(*)*p*<0.1,

* *p*<0.05,

** *p*<0.01,

*** *p*≤0.001.

### Species Richness in Countries

All tested environmental variables showed some significant effects on species richness within countries (Table 7). With respect to productivity-diversity relationships, species richness of spiders, beetles, bugs and all groups combined increased with *NPP*. The ambient energy-models revealed increased species richness of beetles, bugs, ants, herbivores, carnivores and all groups combined with *T*. However, for all groups combined this relationship was no longer significant when an overly influential data point (Portugal) was omitted from the analysis. As for metabolic theory, the slope of log_e_ (species richness) versus *T* ^−1^ came close to the predicted value of −0.65 for ants (−0.64±0.38 95% CI), but was shallower in the remaining cases (all groups: −0.36±0.29 95% CI, spiders: −0.21±0.29 95% CI, beetles: −0.38±0.32 95% CI, bugs: −0.36±0.26 95% CI, herbivores: −0.34±0.29 95% CI, carnivores: −0.38±0.30 95% CI).

**Table 7:** Models of species richness in *N* = 25 European countries.

	Group	*I*-lm	*I*-sar	*AICc*	*r* ^2^	Area	*NPP*	*NPP* ^2^	*T*	*R*	*PET_min_*	*PET_min_* ^2^	*T_range_*	*NPP_range_*	*R_range_*	*PET_minrange_*
PDR	All	0.21	−0.01	429.84	0.61	1101.80***	835.03**		NA	NA	NA	NA	NA	NA	NA	NA
	Spiders	0.32*	0.01	335.28	0.67	165.13***	139.61***		NA	NA	NA	NA	NA	NA	NA	NA
	Beetles	0.12	0.01	405.16	0.57	569.76***	525.00***		NA	NA	NA	NA	NA	NA	NA	NA
	Bugs	0.30*	−0.02	358.80	0.62	240.80***	169.84**		NA	NA	NA	NA	NA	NA	NA	NA
	Ants	0.50***	−0.05	274.10	0.53	32.74***	22.49		NA	NA	NA	NA	NA	NA	NA	NA
	Herbi	0.64***	0.10	398.67	0.67	690.92***			NA	NA	NA	NA	NA	NA	NA	NA
	Carni	0.57***	0.01	380.98	0.59	454.75***			NA	NA	NA	NA	NA	NA	NA	NA
AE	All^a^	0.41**	−0.05	431.98	0.58	952.35***	NA	NA	672.35*	NA	NA	NA	NA	NA	NA	NA
	Spiders	0.59***	0.22	340.00	0.55	165.33***	NA	NA		NA	NA	NA	NA	NA	NA	NA
	Beetles^b^	0.40**	−0.04	406.66	0.54	539.99***	NA	NA	412.98*	NA	NA	NA	NA	NA	NA	NA
	Bugs^c^	0.40*	−0.07	359.23	0.61	221.69***	NA	NA	182.47**	NA	NA	NA	NA	NA	NA	NA
	Ants	0.01	−0.01	270.27	0.59	28.56***	NA	NA	44.22***	NA	NA	NA	NA	NA	NA	NA
	Herbi	0.54***	0.07	398.64	0.71	659.08***	NA	NA	333.68*	NA	NA	NA	NA	NA	NA	NA
	Carni	0.48***	−0.01	380.89	0.64	427.61***	NA	NA	238.79*	NA	NA	NA	NA	NA	NA	NA
IGM	All	0.66***	0.10	426.16	0.71	819.48***	NA	NA	NA		1720.65***	−1743.88***	NA	NA	NA	NA
	Spiders	0.26*	0.08	340.59	0.60	156.28***	NA	NA	NA	−80.87*			NA	NA	NA	NA
	Beetles	0.63***	0.12	400.71	0.69	451.61***	NA	NA	NA		1154.14***	−1111.69***	NA	NA	NA	NA
	Bugs	0.68***	0.05	354.88	0.72	196.31***	NA	NA	NA		386.59**	−395.98***	NA	NA	NA	NA
	Ants^d^	0.60***	0.07	266.59	0.70	24.04***	NA	NA	NA		66.79***	−75.90***	NA	NA	NA	NA
	Herbi	0.68***	0.22	397.09	0.76	553.13***	NA	NA	NA		837.70**	−748.06**	NA	NA	NA	NA
	Carni	0.65***	0.10	380.22	0.70	361.16***	NA	NA	NA		542.490**	−527.20**	NA	NA	NA	NA
SEH	All	0.41**	−0.10	425.40	0.68	482.12***	NA	NA	NA	NA	NA	NA	1048.14***			
	Spiders	0.45**	−0.04	333.17	0.70	76.60*	NA	NA	NA	NA	NA	NA	167.24***			
	Beetles	0.34*	−0.08	401.78	0.62	263.72	NA	NA	NA	NA	NA	NA	608.02**			
	Bugs	0.46**	−0.08	355.29	0.67	128.03*	NA	NA	NA	NA	NA	NA	224.74**			
	Ants	0.55***	−0.08	262.26	0.71	6.16	NA	NA	NA	NA	NA	NA	51.81***			
	Herbi	0.50***	−0.06	393.92	0.76	406.07***	NA	NA	NA	NA	NA	NA	524.83**			
	Carni	0.41**	−0.07	379.69	0.67	297.40***	NA	NA	NA	NA	NA	NA	284.22*			
Best	All	−0.13	−0.08	415.31	0.87	420.67*	801.67***		267.26**	288.08**			796.89***			
	Spiders	−0.12	−0.08	326.40	0.83	110.77**	146.04***			77.56***			118.30***			
	Beetles	−0.25	−0.10	393.00	0.80	247.10	520.40***			229.17***			424.57***			
	Bugs	−0.09	0.01	344.61	0.84	82.76*	160.10***		145.35***				209.83***			
	Ants	−0.37	−0.06	239.63	0.91	−10.03			45.11***	−9.52**			47.26***			
	Herbi	−0.08	−0.07	387.77	0.88	238.01**	445.08***		181.90**	168.41**			549.05***			
	Carni	−0.10	−0.01	372.75	0.81	97.03	316.28***		157.14**				334.71***			

Abbreviations: PDR = productivity-diversity relationships, AE = ambient energy, IGM = interim general model, SEH = spatial environmental heterogeneity, Herbi = herbivores, Carni = carnivores. “NA” indicates cells that were excluded from the respective models. Empty cells were considered, but the respective explanatory variable did not remain in the model according to the second order Akaike Information Criterion (*AICc*). *I*-lm denotes Moran’s *I* correlation coefficient for the residuals of a linear regression model not corrected for spatial autocorrelation. *I*-sar denotes the respective Moran’s *I* for spatial autoregressive error models. *AICc* and *r* ^2^ values are from the spatial regression models, whereby *r* ^2^ is Nagelkerke’s pseudo *r* ^2^ based on maximum likelihood. Values in the columns of environmental variables are model coefficients, with significance levels denoted by asterisks:

* *p*<0.05, ** *p*<0.01, *** *p*<0.001.

a Cook’s distance was >0.5 for Portugal. When excluded, *T* no longer remained in the model.

b Cook’s distance was >0.5 for Portugal. When excluded, the slope for *T* became slightly steeper (434.37*).

c Cook’s distance was >0.5 for Portugal. When excluded, the slope for *T* became slightly flatter (153.17*).

d Cook’s distance was >0.5 for Portugal. When excluded, none of the *IGM* variables remained in the model.

With respect to biological relativity to water-energy dynamics, the full interim general model including both variables (*R* and linear and quadratic terms of *PET_min_*) had constantly higher *AICc* values than simplified models (Table 7). The reduced interim general model for spiders revealed an unexpected negative response of species richness to rainfall (Table 7). The remaining groups showed hump-shaped relationships with *PET_min_* in accordance with biological relativity to water-energy dynamics. Portugal was overly influential in the interim general model for ants, and no significant model remained after its removal. There were consistent positive relationships of arthropod richness with spatial environmental heterogeneity (Table 7). *T_range_* gave a better model fit than *NPP_range_*, *R_range_* and *PET_minrange_* in all cases.

Models with free variable selection always combined variables from several theories (Table 7). They were statistically superior to any single theory according to their higher explanatory power and lower *AICc* values (Δ*AICc* >6.1). Residuals showed significant spatial autocorrelation in the majority of single-theory models, but in none of the models with free variable selection (Table 7, “Best”). This suggests that the models with free variable selection included the majority of relevant variables while single theories tended to miss crucial information. In accordance with a high importance of spatial environmental heterogeneity, species richness increased with *T_range_* in all models with free variable selection (Table 7).

## Discussion

Although our results from the local habitat samples were variable with respect to environmental effects on species richness, there were numerous significant effects of environmental variables on species richness of the same groups at the country grain. This suggests that productivity-diversity relationships, ambient energy, the interim general model and spatial environmental heterogeneity all contribute to the explanation of arthropod species richness of European countries. However, model selection according to *AICc* identified *T_range_* as the strongest predictor of arthropod richness across all studied groups. Independent support for a strong role of spatial heterogeneity in *T* comes from the significant relationship of species turnover across locations with differences in *T*. If species turnover across locations is driven by *T*, then countries with a high *T_range_* will contain higher beta diversity and consequently more species in total than countries with more uniform temperatures. In the following, we will discuss the different theories for broad-scale gradients in species richness and what can be concluded from our data.

### Productivity-diversity Relationships

The observed increase of species richness in countries with *NPP* is in accordance with the majority of studies on broad-scale relationships of species richness with climate [5]. In contrast, relationships of species richness with *NPP* at the local grain were hump-shaped and restricted to carnivores and to the sum of all arthropod species. This accords with a generally reduced effect size [51], and with a transition from monotonous to hump-shaped productivity-diversity relationships towards small spatial grain [7]. The differences between grains cannot be explained by differences in gradient length, because *NPP* varied only slightly more among locations than among countries (locations: 0.39 g C m^−2^ a^−1^ in Lesvos to 0.66 g C m^−2^ a^−1^ near Kraków; countries: 0.41 g C m^−2^ a^−1^ in Norway to 0.65 g C m^−2^ a^−1^ in France; Tables 1,2). Nevertheless, the *NPP* gradient was relatively short at both grains. When gradients include areas with very low *NPP*, stronger effects at the local grain would be expected. Thus, any conclusions with respect to small-scale productivity-diversity relationships from our data should be made with caution.

### Ambient Energy

We found significant effects of *T* on species richness at both local and country grains. The increase of ant species richness with *T* in both near-natural local habitats and in countries accords with ambient energy theories. In contrast, spider richness increased with *T* in intensive agricultural habitats but not at the country grain. This suggests that the richness pattern of spiders in agricultural habitats is not indicative of other habitat types and thus of limited relevance for their overall species richness in countries. Beetles, bugs, carnivores and herbivores showed significant positive relationships with *T* at the country, but not at the local grain. *T* can influence species via its effect on *NPP*. Low temperatures limit terrestrial *NPP* in temperate to arctic climates [5], and large parts of our study area lie in the temperate to boreal region. However, the latitudinal gradient of *NPP* was unimodal in our study, with decreasing *NPP* from temperate to Mediterranean climate (Fig. 1A). At the country scale, all groups except spiders and beetles were significantly affected by *T* in addition to *NPP*, suggesting direct effects of ambient energy on species richness. Across countries, the slope of log_e_(species richness) versus *T* ^−1^ accorded with metabolic theory only for ants. Species richness of ants has been found earlier to conform with metabolic theory [12], [52]. Our results suggest that this may be an exception rather than the rule among terrestrial arthropods. Based on widespread nonlinearity, geographic and taxonomic dependence of temperature-richness relationships, metabolic theory has been more generally questioned [46]. Deviations from metabolic theory can be due to violations of its assumptions [53]. For example, the assumption of body size invariance with temperature is violated by the significant increase of spider body size across Europe with temperature [54]. Tests of tropical niche conservatism and dispersal limitation after glaciation require phylogenetic analyses that exceed the scope of the current investigation [13], [14]. These historical climatic explanations predict more basal taxa in warm climates and high richness of few derived taxa in cooler climate. Thus, the richness of higher taxonomic categories such as families should increase more strongly towards warm climate than the number of species. Such a pattern is present in our spider data: while species richness shows no significant relationship with ambient energy at the country grain, the number of spider families increases with *T* across countries (*t_1,23_* = 4.9, *p*<0.001). This indicates niche conservatism in warmer climates and encourages more detailed phylogenetic exploration of the distribution of European arthropods.

### Temperature, Species Turnover and Spatial Environmental Heterogeneity

In contrast to its variable effect on species richness within local habitats (alpha diversity), differences in *T* had strong effects on species turnover across locations (beta diversity; Table 6). This role of *T* confirms that it represents an important niche dimension of European arthropods [24], [25]. The number of available niches in a given area thus correlates to the range of temperatures present in that area. Our results are consistent with the ideas that species richness is enhanced by (i) elevational range and (ii) habitat heterogeneity in an area [19], [55], [56]. Elevation is a main driver of temperature variation in mountains, leading to correlations between elevational range and spatial heterogeneity in temperature (*r* = 0.92 for the countries studied here). The occurrence of the same ecosystems at similar temperatures across the world that have contrasting elevations demonstrates that temperature is more crucial for biodiversity than elevation *per se* [57]. Being difficult to measure, habitat heterogeneity is often determined by the number of distinguishable vegetation types present in an area [56]. In near-natural situations, vegetation types are in turn determined by the environmental preferences of their constituent plant species, including their temperature preference [58]. Thus, spatial environmental heterogeneity, elevational range and habitat heterogeneity are interrelated, and we presume that climate often has the most direct influence on biodiversity. In our study, variability in *T* had a dominant effect on species turnover and gamma diversity. Apart from the existence of more niches along large temperature gradients, climatic heterogeneity can also buffer species extinctions by allowing species confronted with climatic fluctuations to relocate to suitable climatic refuges [59]. The dominant role of *T_range_* for arthropod species richness in European countries is in accordance with both enhanced niche availability and reduced extinction during climatic fluctuations.

### Biological Relativity to Water-energy Dynamics

Biological relativity to water-energy dynamics is expressed in the interim general model [16], [17]. It predicts increasing species richness with *R* (water term) and a unimodal relationship of species richness with *PET_min_* (energy term). We found only partial support for IGM, since reduced models always resulted in lower *AICc* values compared to the full model (Tables 5,7). Numbers of spider species per country decreased with *R* in the interim general model, but increased with *R* in the model with free variable selection. Dominant effects of the energy term in the interim general models at the country grain were replaced by group-specific positive, negative (ants) or absent (herbivores) effects of *R* in the models with free variable selection (Table 7). Given these inconsistencies, the interim general model provided no robust explanation of arthropod richness in our study. Nevertheless, the numerous significant relationships with *R* and *PET_min_* suggest that the interim general model may apply to European arthropods, but that larger datasets are necessary to disentangle its components. The dominant effect of the water term in the models with free variable selection is in accordance with Hawkins et al. [18], who found that the energy term of the interim general model becomes dispensable in temperate to arctic climate.

### Herbivores versus Carnivores

The strength of latitudinal diversity gradients has been found to increase across trophic levels [51]. In our study, the major difference between herbivores and carnivores was at the local grain, where carnivores showed a hump-shaped relationship to *NPP* and herbivores no significant relationship at all. At the country grain, the results for herbivores and carnivores were similar to each other and to those of bugs and all arthropods combined. This suggests that the observed differences between arthropod taxa are due to their different phylogeny or other life-history traits rather than caused by their trophic position.

### Countries versus Local Habitats

The stronger and more consistent effects in countries versus locations are in accordance with the general decrease of species-richness environment relationships towards small spatial grain [51], [56], [60]. Ecological processes are scale-dependent [61], and effects of the studied broad-scale environmental conditions may affect regional species pools rather than local assemblages. However, species pools can affect local species richness [62], especially in mobile organisms such as the studied arthropod groups. In addition, some of the mechanisms to explain broad-scale patterns in species richness include local processes such as resource partitioning (productivity-diversity relationships), metabolism (metabolic theory), or cold tolerance (tropical niche conservatism). Thus, contrasts such as the variable role of ambient energy at locations and its widespread positive effect across countries are remarkable and provide a starting point for further research.

Differences in data quality could have contributed to the stronger effects in countries versus locations [10]. First, our pitfall traps sampled only ground-dwelling arthropods over a limited time period. The 33223 sampled individuals of 1066 species already represent a major identification effort. Nevertheless, sampling intensity per habitat corresponded only to a minimal effort that is expected to encompass around 75% of all species attainable at the respective site with pitfall traps [63]. Even more problematic can be differences in sampling efficiency between sites due, for example, to weather or habitat structure [64]. We reduced these differences by applying strictly standardized sampling methods and by including the number of individuals captured per habitat as a factor in the models of species richness. By using (log) individual numbers as a factor, we assume that true abundances are similar across habitats and that observed differences in individual numbers are due to variation in sampling efficiency. However, true abundances may differ. Results at the local grain would change strongly if individual numbers were excluded from the models – the only consistency being increased ant richness with ambient energy and variable effects between groups (results not shown). This highlights that the difficulty to obtain large, representative arthropod samples from defined areas remains a main obstacle in community ecology. Accordingly, conclusions from the results at the local grain should be drawn with care. In contrast to these sampling issues at the local grain, species inventories of countries are the results of many decades of research and have reached asymptotes in almost all cases [65]. A second difference in data quality between locations and countries relates to the environmental data. Interpolated climatic variables will result in relatively accurate values across large areas such as countries, but have only limited accuracy at the grain of local habitats. Sources of error include spatially and temporally unpredictable factors such as rainfall, as well as anthropogenic effects on productivity, especially in disturbed habitats. Thus, sampling error at the locations is probably much higher than at the country grain, underlining the need for additional high-quality inventories of invertebrates along environmental gradients of large spatial extent.

## Main Conclusions

Our study supports the scale-dependence of species richness-environment relationships. While relationships of local species richness with environmental variables were contingent on the arthropod group, species richness patterns at the country grain were more consistent and partly supported all tested theories. Niche theory provides a plausible link between the two grains: On the one hand, differences in temperature were the best correlate of species turnover across locations. On the other hand, spatial heterogeneity in annual mean temperature had the strongest effects on arthropod diversity within European countries. These two independent findings suggest that temperature is an important niche dimension and that countries with wider ranges in annual mean temperature provide a greater breadth of niche space and so can support larger numbers of arthropod species. Unless environmental heterogeneity is constant across sampling units (thinkable e.g. in marine environments), we strongly suggest that studies with large sampling units take into account environmental heterogeneity, just as studies with variable area of sampling units nowadays routinely consider area [49].

## References

[1] Rosenzweig ML, Abramski Z (1993) How are diversity and productivity related? In: University of Chicago Press, Chicago Ricklefs RE, Schluter C, editors. Species diversity in ecological communities: historical and geographical perspectives. IL: pp. 52–65.

[2] Abrams PA (1995) Monotonic or unimodal diversity-productivity gradients: what does competition theory predict? Ecology 76: 2019–2027.

[3] Mittelbach GG, Steiner CF, Scheiner SM, Gross KL, Reynolds HL, et al (2001) What is the observed relationship between species richness and productivity? Ecology 82: 2381–2396.

[4] Cardinale BJ, Hillebrand H, Harpole WS, Gross K, Ptacnik R (2009) Separating the influence of resource ‘availability’ from resource ‘imbalance’ on productivity-diversity relationships. Ecol Lett 12: 475–487. 19490011 10.1111/j.1461-0248.2009.01317.x

[5] Hawkins BA, Field R, Cornell HV, Currie DJ, Guégan J-F, et al (2003) Energy, water, and broad-scale geographic patterns of species richness. Ecology 84: 3105–3117.

[6] Lavers C, Field R (2006) A resource-based conceptual model of plant diversity that reassesses causality in the productivity–diversity relationship. Global Ecol Biogeogr 15: 213–224.

[7] Chase JM, Leibold MA (2002) Spatial scale dictates the productivity-biodiversity relationship. Nature 416: 427–430. 11919631 10.1038/416427a

[8] Willig MR, Kaufman DM, Stevens RD (2003) Latitudinal gradients of biodiversity: pattern, process, scale, and synthesis. Annu Rev Ecol Evol Syst 34: 273–309.

[9] Evans KL, Warren PH, Gaston KJ (2005) Species-energy relationships at the macroecological scale: a review of the mechanisms. Biol Rev 80: 1–25. 15727036 10.1017/s1464793104006517

[10] Whittaker RJ (2010) Meta-analyses and mega-mistakes: calling time on meta-analysis of the species richness–productivity relationship. Ecology 91: 2522–2533. 20957942 10.1890/08-0968.1

[11] Allen AP, Brown JH, Gillooly JF (2002) Global biodiversity, biochemical kinetics, and the energetic-equivalence rule. Science 297: 1545–1548. 12202828 10.1126/science.1072380

[12] Kaspari M, Ward PS, Yuan M (2004) Energy gradients and the geographic distribution of local ant diversity. Oecologia 140: 407–413. 15179582 10.1007/s00442-004-1607-2

[13] Hawkins BA (2010) Multiregional comparison of the ecological and phylogenetic structure of butterfly species richness gradients. J Biogeogr 37: 647–656.

[14] Hortal J, Diniz-Filho JAF, Bini LM, Rodríguez MÁ, Baselga A, et al (2011) Ice age climate, evolutionary constraints and diversity patterns of European dung beetles. Ecol Lett 14: 741–748. 21645193 10.1111/j.1461-0248.2011.01634.x

[15] Brown JH, Gillooly JF, Allen AP, Savage VM, West GB (2004) Toward a metabolic theory of ecology. Ecology 85: 1771–1789.

[16] Field R, O’Brien EM, Whittaker RJ (2005) Global models for predicting woody plant richness from climate: development and evaluation. Ecology 86: 2263–2277.

[17] O’Brien EM (2006) Biological relativity to water-energy dynamics. J Biogeogr 33: 1868–1888.

[18] Hawkins BA, Montoya D, Rodríguez MÁ, Olalla-Tárraga MÁ, Zavala MÁ (2007) Global models for predicting woody plant richness from climate: comment. Ecology 88: 255–259. 17489474 10.1890/0012-9658(2007)88[255:gmfpwp]2.0.co;2

[19] Rahbek C (2005) The role of spatial scale and the perception of large-scale species-richness patterns. Ecol Lett 8: 224–239.

[20] Hutchinson GE (1958) Concluding remarks. In: Cold Spring Harbour Symposia on Quantitative Biology 22: 415–427.

[21] Schoener TW (1989) The ecological niche. In: Cherrett JM, editor. Ecological concepts. Blackwell, Oxford. pp. 79–114.

[22] Thomas CD, Cameron A, Green RE, Bakkenes M, Beaumont LJ, et al (2004) Extinction risk from climate change. Nature 427: 145–148. 14712274 10.1038/nature02121

[23] Hill MO, Roy DB, Mountford JO, Bunce RGH (2000) Extending Ellenberg’s indicator values to a new area: an algorithmic approach. J Appl Ecol 37: 3–15.

[24] Eyre MD, Rushton SP, Luff ML, Telfer MG (2005) Investigating the relationships between the distribution of British ground beetle species (Coleoptera: Carabidae) and temperature, precipitation and altitude. J Biogeogr 32: 973–983.

[25] Entling W, Schmidt MH, Bacher S, Brandl R, Nentwig W (2007) Niche properties of Central European spiders: shading, moisture, and the evolution of the habitat niche. Global Ecol Biogeogr 16: 440–448.

[26] Hawkins BA, Diniz-Filho JAF, Jaramillo CA, Soeller SA (2006) Post-Eocene climate change, niche conservatism, and the latitudinal diversity gradient of New World birds. J Biogeogr 33: 770–780.

[27] Koleff P, Gaston KJ (2002) The relationships between local and regional species richness and spatial turnover. Global Ecol Biogeogr 11: 363–375.

[28] Fauna Europaea Web Service (2004) Fauna Europaea version 1.1. Available: http://www.faunaeur.org. Accessed 2008.

[29] Hammen VC, Biesmeijer JC, Bommarco R, Budrys E, Christensen TR, et al (2010) Establishment of a cross-European field site network in the ALARM project for assessing large-scale changes in biodiversity. Environ Monit Assess 164: 337–348. 19365607 10.1007/s10661-009-0896-7

[30] Settele J, Hammen VC, Hulme P, Karlson U, Klotz S, et al (2005) ALARM: Assessing LArge-scale environmental Risks for biodiversity with tested Methods. GAIA 14: 69–72.

[31] Rötzer T, Chmielewski FM (2001) Phenological maps of Europe. Climate Res 18: 249–257.

[32] Mitchell TD, Carter TR, Jones PD, Hume M, New M (2004) A comprehensive set of high-resolution grids of monthly climate for Europe and the globe: the observed record (1901–2000) and 16 scenarios (2001–2100) Tyndall Center, University of East Anglia, Norwich.

[33] New M, Hulme M, Jones P (2000) Representing twentieth-century space-time climate variability Part II: development of 1901–96 monthly grids of terrestrial surface climate. J Climate 13: 2217–2238.

[34] Smith B, Prentice IC, Sykes MT (2001) Representation of vegetation dynamics in the modelling of terrestrial ecosystems: comparing two contrasting approaches within European climate space. Global Ecol Biogeogr 10: 621–637.

[35] Hickler T, Smith B, Sykes MT, Davis M, Sugita S, et al (2004) Using a generalized vegetation model to simulate vegetation dynamics in northeastern USA. Ecology 85: 519–530.

[36] Hickler T, Fronzek S, Araújo MB, Schweiger O, Thuiller W, et al (2009) An ecosystem-model-based estimate of changes in water availability differs from water proxies that are commonly used in species distribution models. Global Ecol Biogeogr 18: 304–313.

[37] Hickler T, Vohland K, Feehan J, Miller PA, Smith B, et al (2012) Projecting the future distribution of European potential natural vegetation zones with a generalized, tree species-based dynamic vegetation model. Global Ecol Biogeogr 21: 50–63.

[38] Zaehle S, Smith B, Hatterman F (2005) Effects of parameter uncertainties on the modelling of terrestrial biosphere dynamics. Global Biogeochem Cy 19: GB3020.

[39] Hickler T, Prentice IC, Smith B, Sykes MT, Zaehle S (2006) Implementing plant hydraulic architecture within the LPJ Dynamic Global Vegetation Model. Global Ecol Biogeogr 15: 567–577.

[40] Smith B, Knorr W, Widlowski J-L, Pinty B, Gobron N (2008) Combining remote sensing data with process modelling to monitor boreal conifer forest carbon balances. Forest Ecol Manage 255: 3985–3994.

[41] Thornthwaite CW (1948) An approach toward a rational classification of climate. Geogr Rev 38: 55–94.

[42] R Development Core Team (2010) R: a language and environment for statistical computing. R Foundation for Statistical Computing, Vienna. Available: http://www.r-project.org. Accessed 2010.

[43] Dormann CF, McPherson JM, Araujo MB, Bivand R, Bolliger J, et al (2007) Methods to account for spatial autocorrelation in the analysis of species distributional data: a review. Ecography 30: 609–628.

[44] Kissling WD, Carl G (2008) Spatial autocorrelation and the selection of simultaneous autoregressive models. Global Ecol Biogeogr 17: 59–71.

[45] Bivand R (2011) spdep: Spatial dependence: weighting schemes, statistics and models. R package version 0.5–35. Available: http://cran.r-project.org/package=spdep. Accessed 2011.

[46] Hawkins BA, Albuquerque FS, Araújo MB, Beck J, Bini LM, et al (2007) Global evaluation of metabolic theory as an explanation for terrestrial species richness gradients. Ecology 88: 1877–1888. 17824415 10.1890/06-1444.1

[47] Anderson MJ, Crist TO, Chase JM, Vellend M, Inouye BD, et al (2011) Nagivating the multiple meanings of β diversity: a roadmap for the practicing ecologist. Ecol Lett 14: 19–28. 21070562 10.1111/j.1461-0248.2010.01552.x

[48] Oksanen J, Blanchet FG, Kindt R, Legendre P, O’Hara RB, et al. (2010). vegan: Community Ecology Package. R package version 1.17–2. Available: http://cran.r-project.org/package=vegan. Accessed 2010.

[49] Rosenzweig ML (1995) Species diversity in space and time. Cambridge University Press, Cambridge.

[50] Kumschick S, Schmidt-Entling MH, Bacher S, Hickler T, Espadaler X, et al (2009) Determinants of local ant (Hymenoptera: Formicidae) species richness and activity density across Europe. Ecol Entomol 34: 748–754.

[51] Hillebrand H (2004) On the generality of the latitudinal diversity gradient. Am Nat 163: 192–211. 14970922 10.1086/381004

[52] Kaspari M (2004) Using the metabolic theory of ecology to predict global patterns of abundance. Ecology 85: 1800–1802.

[53] Cassemiro FAS, Diniz-Filho JAF (2010) Deviations from predictions of the metabolic theory of ecology can be explained by violations of assumptions. Ecology 91: 3729–3738. 21302843 10.1890/09-1434.1

[54] Entling W, Schmidt-Entling MH, Bacher S, Brandl R, Nentwig W (2010) Body size-climate relationships of European spiders. J Biogeogr 37: 477–485.

[55] Finch O-D, Blick T, Schuldt A (2008) Macroecological patterns of spider species richness across Europe. Biodiv Conserv 17: 2849–2868.

[56] Belmaker J, Jetz W (2011) Cross-scale variation in species richness-environment associations. Global Ecol Biogeogr 20: 464–474.

[57] Begon M, Townsend CR, Harper JL (2006) Ecology: from individuals to ecosystems. Blackwell, Malden.

[58] Ellenberg H, Leuschner C (2010) Vegetation Mitteleuropas mit den Alpen in ökologischer, dynamischer und historischer Sicht. Ulmer, Stuttgart.

[59] Ohlemüller R, Anderson BJ, Araújo MB, Butchart SHM, Kudrna O, et al (2008) The conicidence of climatic and species rarity: high risk to small-range species from climatic change. Biol Lett 4: 568–572. 18664421 10.1098/rsbl.2008.0097 PMC2610064

[60] Field R, Hawkins BA, Cornell HV, Currie DJ, Diniz-Filho JAF, et al (2009) Spatial species-richness gradients across scales: a meta-analysis. J Biogeogr 36: 132–147.

[61] Wiens JA (1989) Spatial scaling in ecology. Funct Ecol 3: 385–397.

[62] Zobel M (1997) The relative of species pools in determining plant species richness: an alternative explanation of species coexistence? Trends Ecol Evol 12: 266–269. 21238064 10.1016/s0169-5347(97)01096-3

[63] Duelli P, Obrist MK, Schmatz DR (1999) Biodiversity evaluation in agricultural landscapes: above-ground insects. Agric Ecosyst Environ 74: 33–64.

[64] Topping CJ, Sunderland KD (1992) Limitations in the use of pitfall traps in ecological studies exemplified by a study of spiders in a field of winter wheat. J Appl Ecol 29: 485–491.

[65] Schuldt A, Assmann T (2010) Invertebrate diversity and national responsibility for species conservation across Europe – A multi-taxon approach. Biol Conserv 143: 2747–2756.

[66] Henle K, Kunin WE, Schweiger O, Schmeller DS, Grobelnik V, et al (2010) Securing the conservation of biodiversity across administrative levels and spatial, temporal, and ecological scales – research needs and approaches of the SCALES project. GAIA 19: 187–193.

